# Technical note: Evaluation of a silicone‐based custom bolus for radiation therapy of a superficial pelvic tumor

**DOI:** 10.1002/acm2.13538

**Published:** 2022-01-27

**Authors:** Karissa M. Wang, Amanda J. Rickards, Trevor Bingham, Jonathan D. Tward, Ryan G. Price

**Affiliations:** ^1^ Department of Biomedical Engineering University of Utah Salt Lake City Utah USA; ^2^ Department of Chemistry Weber State University Ogden Utah USA; ^3^ Huntsman Cancer Institute Salt Lake City Utah USA; ^4^ Department of Radiation Oncology University of Utah Salt Lake City Utah USA

**Keywords:** 3d‐printed, bolus, custom bolus

## Abstract

**Purpose:**

Use of standard‐of‐care radiation therapy boluses may result in air‐gaps between the target surface and bolus, as they may not adequately conform to each patient's unique topography. Such air‐gaps can be particularly problematic in cases of superficial pelvic tumor radiation, as the density variation may result in the radiation delivered to the target site being inconsistent with the prescribed dose. To increase bolus fit and thereby dose predictability and homogeneity, we designed and produced a custom silicone bolus for evaluation against the clinical standard.

**Methods:**

A custom bolus was created for the pelvic regions of both an anthropomorphic phantom and a pelvic patient with squamous cell carcinoma of the penile shaft. Molds were designed using computed tomography (CT) scans, then 3D‐printed and cast with silicone rubber to yield the boluses. Air‐gap measurements were performed on custom and standard‐of‐care Superflab gel sheet boluses by analyzing total volume between the bolus and target surface, as measured from CT scans. Therapeutic doses of radiation were delivered to both boluses. Radiation dose was measured and compared to the expected dose using nine optically stimulated luminescent dosimeters (OSLDs) placed on the phantom.

**Results:**

Mean air‐gap volume between the bolus and phantom was decreased from 314 ± 141 cm^3^ with the standard bolus to 4.56 ± 1.59 cm^3^ using the custom device. In the case of the on‐treatment patient, air‐gap volume was reduced from 169 cm^3^ with the standard bolus to 46.1 cm^3^ with the custom. Dosimetry testing revealed that the mean absolute difference between expected and received doses was 5.69%±4.56% (15.1% maximum) for the standard bolus and 1.91%±1.31% (3.51% maximum) for the custom device. Areas of greater dose difference corresponded to areas of larger air‐gap.

**Conclusions:**

The custom bolus reduced air‐gap and increased predictability of radiation dose delivered compared to the standard bolus. The custom bolus could increase the certainty of prescribed dose‐delivery of radiation therapy for superficial tumors.

## 1 INTRODUCTION

Radiation therapy (RT) is often used to treat cancers where superficial dose to the skin is of utmost importance. However, the current standard‐of‐care bolus does not conform adequately to each patients’ unique topography, which may result in air‐gaps between the bolus and target surface. Such air‐gaps persist for a range of skin cancer areas and are especially common with tumors present in the pelvic region, where they can cause significant surface dose variations^2^ which could compromise the quality of treatment. Although the exact magnitude of dose error differs depending upon treatment energy, field size, bolus thickness, and air‐gap size, previous research has shown that, in some cases, air‐gaps of 2 cm resulted in relative surface dose measurements more than 30% less than a surface dose without air‐gap.^3^

3D printing has been investigated for use in manufacture of patient‐specific boluses. It has been shown that 3D‐printed boluses are able to provide more accurate dose distribution compared to the current standard‐of‐care.^1^, ^4^, ^5^ However, boluses that are directly 3D‐printed do not conform fully to patients due to the rigid nature of printing material. This has led to the use of 3D printers to create a mold, and subsequently cast a bolus that is created from a softer, more conformable material.^6^, ^7^

This study further investigated the ability of 3D printers to create a custom mold and subsequently cast a conformable, patient‐specific bolus. Specifically, this study evaluated this type of custom bolus in reducing air‐gap in the context of superficial pelvic cancer with complex geometry. In an effort to increase predictability and homogeneity of dose in superficial pelvic cancers, we designed and produced a custom bolus and evaluated it against the clinical standard. This process utilized the standard‐of‐care CT simulation scan to design and 3D print a mold for the custom bolus, which was then cast with silicone rubber to create the final bolus. A bolus that minimizes air‐gap could lead to more accurate radiation dose delivery, resulting in improved patient outcomes.

## 2 METHODS

### 2.1 Mold and bolus generation

Two custom boluses designed for superficial pelvic tumors were generated, one for an on‐treatment patient and the other for a phantom manufactured in‐house. This phantom was created by merging open‐source anatomical standard tessellation language (STL) files provided by Scan the World and Cults3D. This phantom included more anatomical detail in the pelvic region than standard phantoms such as the RANDO phantom. Standard‐of‐care CT scans were used to design the molds, with CT scan parameters of 120 kV, 700 MAs, and 2.5 mm slice thickness and patient scan parameters of 120 kV, 650 mAs, and 2 mm slice thickness. Mold design used open‐source segmentation (Seg3D, version 2.2.1) and tetrahedral mesh generator software (SCIRun, version 5.0) to output an STL file describing the shape and size of the uniform thickness bolus to be manufactured. A lattice‐cleaving algorithm (Cleaver, version 2.4) smoothed the mold surface to generate an accurate fit.

The STL file of the bolus shape was imported into Fusion 360 (version 2.0.7813). The bolus shape was subtracted from a box to create the basic design of the mold. This box design was chosen to make the mold easy to store and transport, in addition to improving the printability. The box mold method allowed the large molds that would not otherwise fit within the 3D printer's print volume to be 3D‐printed in segments which were subsequently combined to form the full mold. The box was then bisected to introduce two halves to the mold. Bolt holes, sprues (small holes designed into the mold to allow air bubbles in the silicone to escape), and a pour hole were added to allow the two halves of the mold to be assembled and cast post‐printing. Additional processing included a handle and clasp that allowed the mold to be easily transported and used as a bolus storage device. Each component of the mold was exported as an STL file, which were sliced using Slic3r (version 2.0.0) and printed on a Prusa i3 MK3S 3D printer with a bed temperature of 85°C for the first layer, 90°C for all other layers, an extruder temperature of 245°C for the first layer, 240°C for all other layers, 20% gyroid infill, and layer height of 0.15 mm, 4 perimeters, 5 bottom solid layers, and 7 top solid layers. Polyethylene terephthalate glycol (PETG) was chosen as the 3D‐printing material due to warp resilience, robustness, and ease of printing and recyclability compared to other plastics of the same price point.

After printing, mold components were assembled and the required printing supports removed. When using the box design, depending on the exact print orientation, supports, raft, and sanding may or may not be necessary. Smooth‐on EcoFlex™ (durometer Shore 00–20) silicone rubber was cast in the mold to create the custom bolus device. This material was chosen for its ease of casting, flexibility, slight tackiness, and density similar to that of water.^8^ Additionally, silicone rubber has been shown to be biocompatible in bolus applications, having several previous uses in this context.^4^, ^5^, ^6^, ^7^ Further, Smooth‐on Ecoflex is marketed as a “skin‐safe” product that may be used in other superficial manners such as prosthetic appliances or orthotic cushioning.^8^ The authors thought that the tackiness of the silicone rubber might help the final device adhere more tightly to the skin, reducing air‐gap volume and accounting for minor day‐to‐day variations in target site morphology such as inflammatory swelling, edema, or tumor regression.

The time required to generate a custom bolus using this method varies depending upon the size of the bolus as well as the size and speed of the 3D printers employed. The patient and phantom boluses described here took ∼1 hour each to design. 3D printing of the phantom bolus mold required ∼2 days and the patient mold required ∼6 days, but timing could be reduced by using a more advanced or newer 3D printer and by employing multiple printers simultaneously. Each mold took half an hour to cast. Using radiochromic film, a range of silicone rubber thicknesses were used to confirm that build‐up properties such as depth of maximum dose (*d*_max_) were similar to that of water. Additional testing was conducted to determine whether build‐up properties of the silicone rubber change after being exposed to therapeutic amounts of radiation exposure.

### 2.2 Air‐gap quantification

Air‐gap measurements were performed on the in‐house phantom and on‐treatment patient using both the standard‐of‐care (Superflab, 1.0 cm and 0.5 cm thickness) and custom boluses. In the standard bolus trials, air‐gaps were “packed” to the best of the radiation therapists’ abilities with additional Superflab, as is standard protocol in the radiation therapy clinic where testing was performed. After the bolus was placed on the phantom or patient, a CT scan was completed at the same standard resolution as the standard‐of‐care pre‐treatment scan. For the patient trial, one CT scan was taken with the standard bolus and another with the custom. The fitting of the patient‐specific bolus, CT scan‐derived measurements, and software methodology were performed under Institutional Review Board approval. With the phantom, 3 CT scans each were taken with standard bolus and the custom bolus. The standard bolus utilized sheets of 1.0 cm thickness and 0.5 cm thickness stacked upon one another to achieve the same thickness as the custom bolus. Between each CT scan, the bolus was removed and re‐placed upon the target site to simulate the variability of setup between sessions. The time taken by radiation therapists to place the custom and standard boluses onto the on‐treatment patient was noted, with start time being when the therapist first touched the bolus and stop time being once therapists were satisfied with final placement.

After CT scans were collected, Seg3D was used to quantify the total air‐gap volume between the bolus and target site using the software's native volume calculation tool to find the total air‐gap volume. The mean of the three trials for each bolus was calculated, as well as the standard deviation of air gap for each bolus. The total air‐gap volume was used as a comparison between the custom bolus and Superflab because it best quantifies the difference between a custom bolus and the current standard‐of‐care.

**FIGURE 1:**
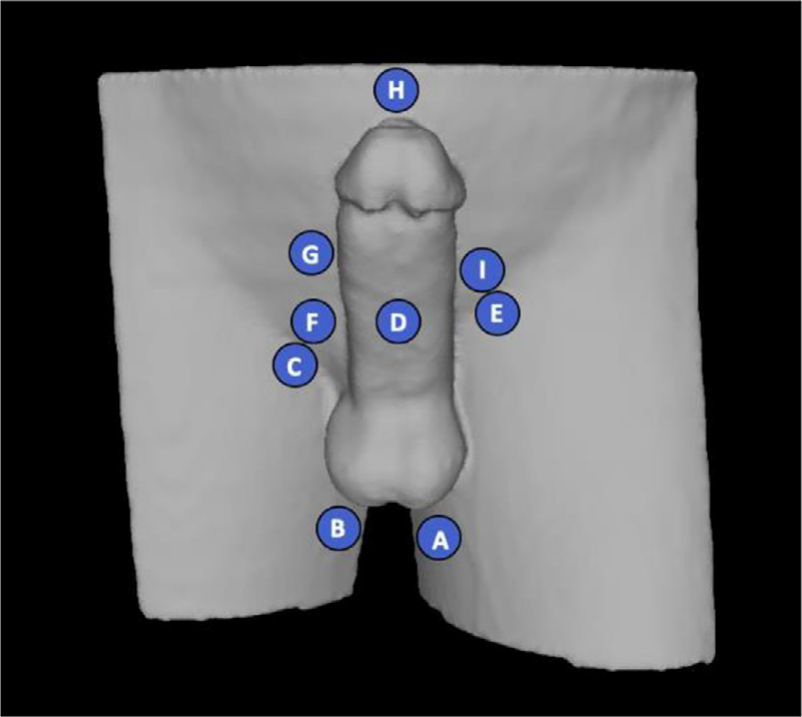
Placement diagram for optically stimulated luminescent dosimeters (OSLDs) on the phantom

The maximum air‐gap heights were also measured for the phantom at the OSLD locations shown in Figure 1. The measurements were taken at the maximum height within 5 mm of the OSLD locations, on all three CT scans. The mean for the maximum air‐gap heights was calculated, as well as the standard deviation of the heights for each bolus.

### 2.3 Dosimetry testing

Depth of max dose was characterized for the custom bolus material relative to water using a parallel plate ionization chamber in a 6 × 6 cm^2^ 6 MV field. 200 MU of radiation were delivered to the in‐house phantom using a 6MV flattened beam for both the standard‐of‐care and custom boluses, each of 1.5 cm thickness. The radiation dose at 9 points on the phantom, shown in Figure 1, was measured using optically stimulated luminescent dosimeters (OSLDs) placed between the bolus and phantom.

The expected dose at each point was calculated by generating a bolus contour of 1.5 cm thickness from the external body contour in the treatment planning software's bolus generation tool (Varian Eclipse, version 15.5) and using the Varian's Acuros algorithm to calculate delivery of 200 MU of radiation from a single 30 × 30 AP field. The actual dose received by each OSLD was compared to the expected dose calculated by the planning software, and the difference between the expected and received dose at each location was calculated for both boluses. Using this information, the mean and standard deviation from expected dose were calculated for the standard‐of‐care and custom boluses. A paired, two‐tail *t*‐test (α = 0.05) was used to evaluate the deviation from the expected dose between the two sample populations.

## 3 RESULTS

### 3.1 Mold and bolus generation

Preliminary testing conducted by the authors showed that maximum radiation build‐up occurred at a silicone rubber thickness of approximately 1.5 cm; thus, subsequent tests were done utilizing bolus of 1.5 cm thickness. Additional testing also showed that the build‐up properties of the silicone rubber do not change after being exposed to therapeutic amounts of radiation exposure.

**FIGURE 2:**
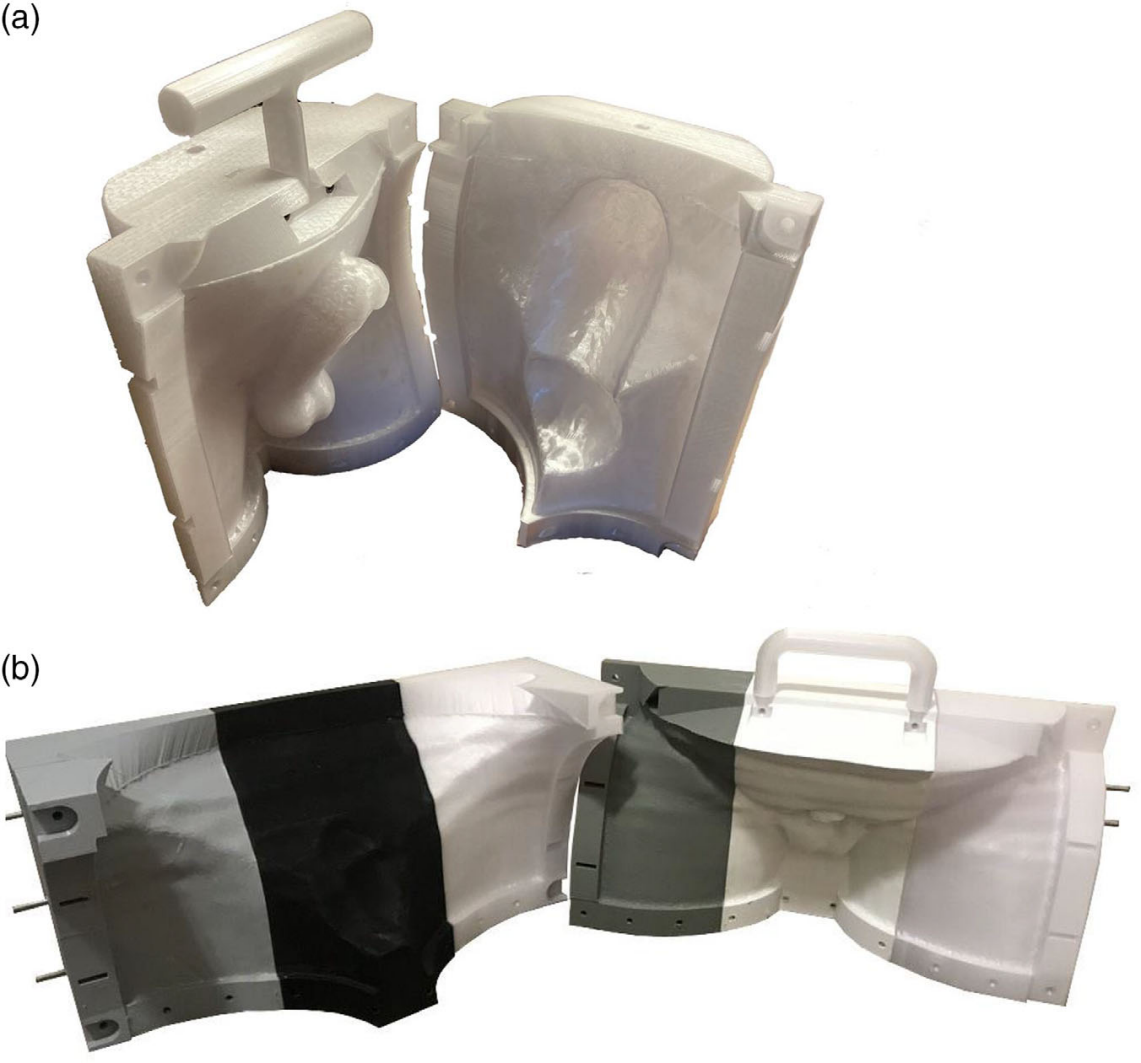
3D‐printed mold to be used to create custom boluses for (a) the phantom and (b) an on‐treatment patient

**FIGURE 3:**
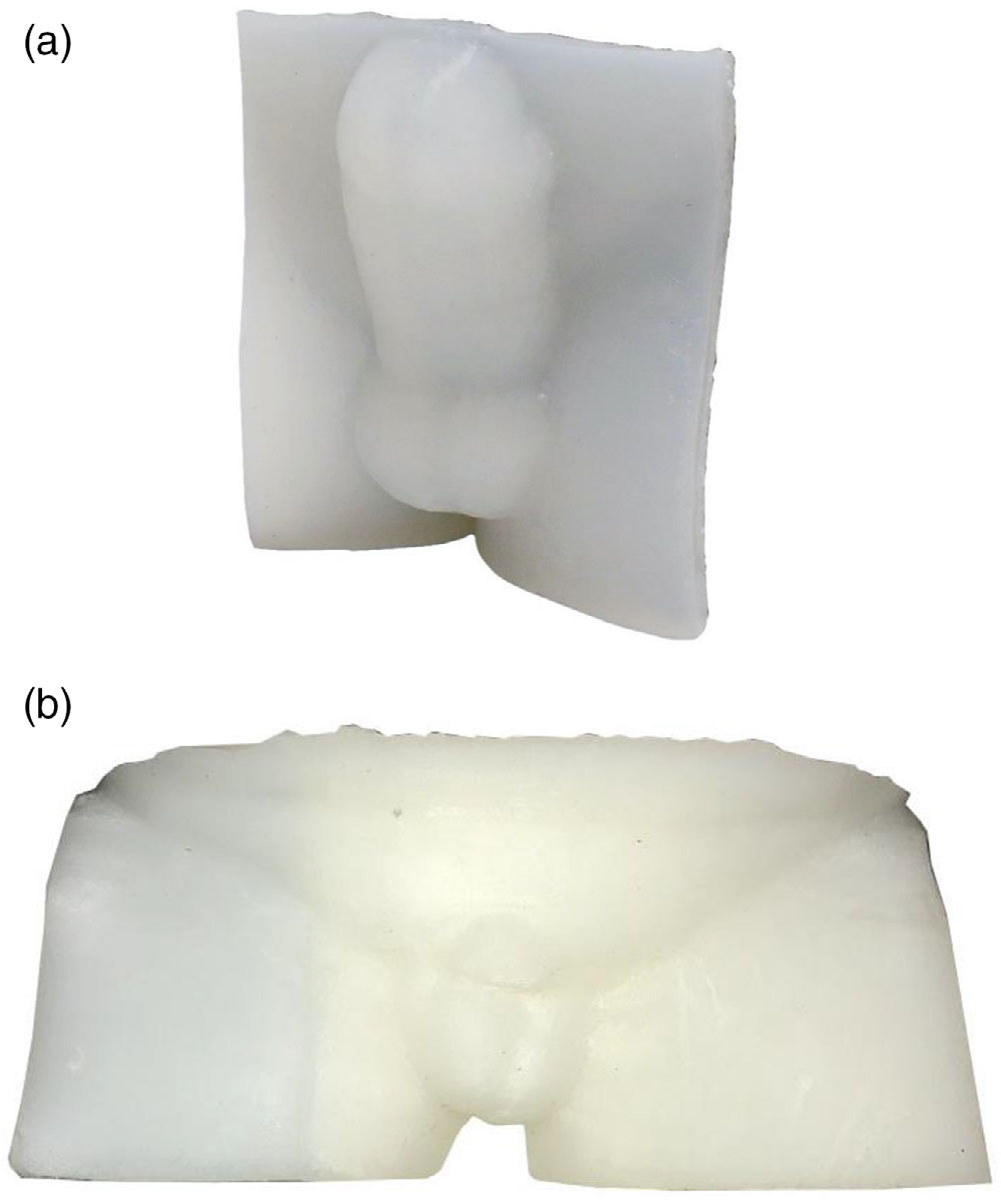
Custom silicone bolus created for (a) the phantom and (b) an on‐treatment patient

Molds for the phantom and patient devices were 3D‐printed and shown in Figure 2. The silicone rubber devices created by casting the molds are shown in Figure 3.

### 3.2 Air‐gap quantification

**FIGURE 4:**
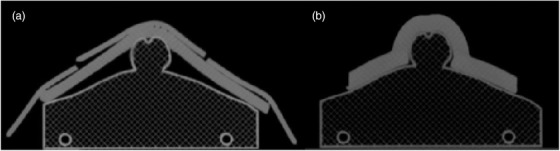
Computed tomography scan of a phantom cross‐section showed visibly reduced air gap using the custom bolus. Cross‐section of (a) standard bolus and (b) custom bolus on the groin of an anthropomorphic phantom

The custom bolus visibly reduced air‐gap between the phantom and bolus, as compared to the standard device, as shown in Figure 4. The phantom had a mean air‐gap volume (*x̄* ±  SD) using the standard bolus of 314 ± 141 cm^3^, this was reduced to 4.56 ± 1.59 cm^3^ using the custom device. In the patient trial, the air‐gap volume was 169 cm^3^ with the standard bolus and 46.1 cm^3^ with the custom device, resulting in a 98.5% reduction in air‐gap volume for the phantom and a 72.7% reduction in the patient trial. The phantom mean air gap height (*x̄* ±  SD) using the standard bolus had a range of 6.27 ±  1.54 mm to 31.70 ±  8.10 mm, reduced to a range of 0.00 ±  0.00 mm to 3.68 ±  0.68 mm by the custom bolus, resulting in an 88.4% reduction in air‐gap height on the phantom.

### 3.3 Dosimetry testing

**FIGURE 5:**
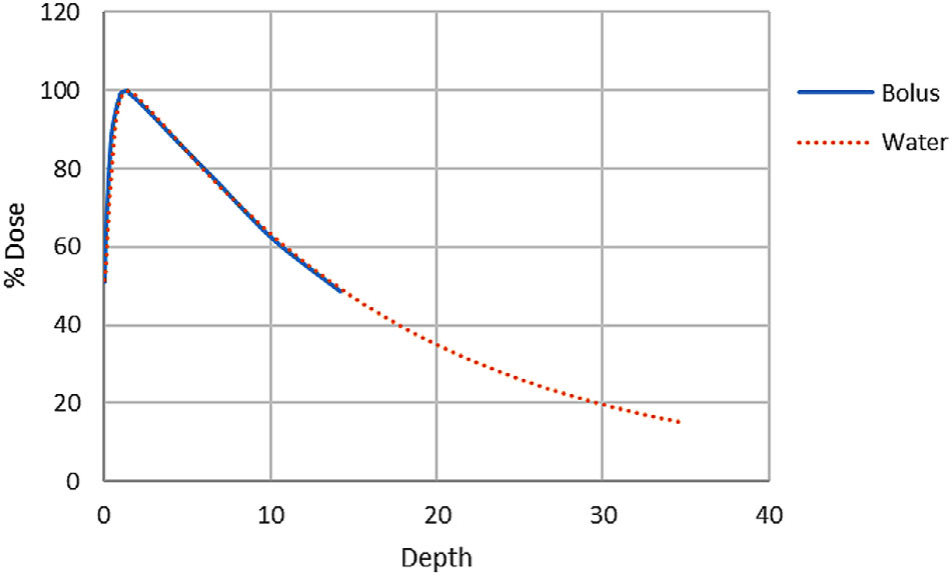
Percent depth dose curves relative to *d*_max_ for both the custom bolus material and water in a 6 × 6 cm^2^ 6 MV photon field

**FIGURE 6:**
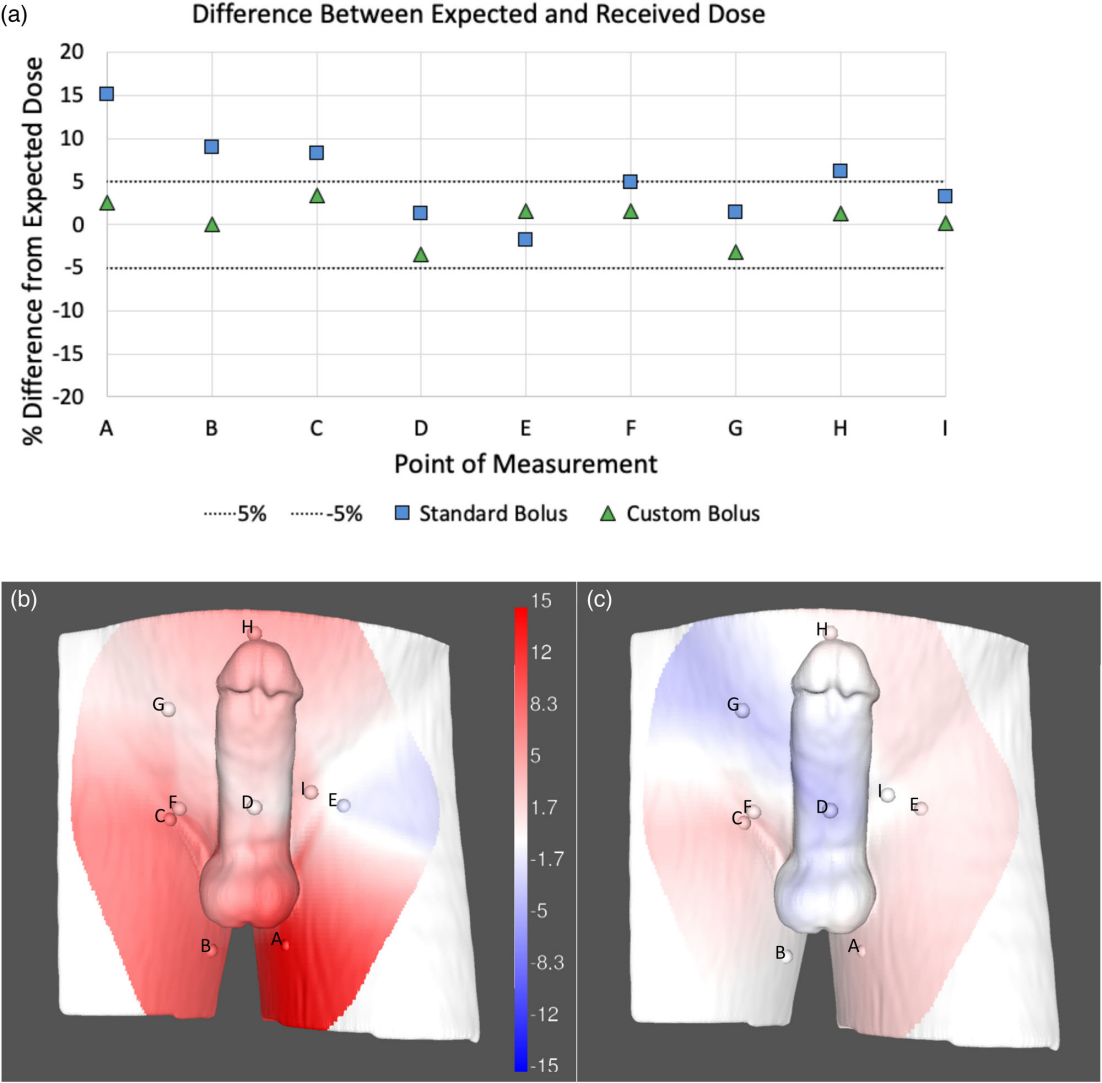
Differences between expected and received radiation dose on a phantom. (a) Percent difference values between measured and predicted radiation dose as measured by optically stimulated luminescent dosimeter (OSLD), (b) visualization of percent difference with standard bolus relative to idealized bolus with no air gaps, as calculated by treatment planning system, (c) visualization of percent difference with custom bolus relative to idealized bolus with no air gaps, as calculated by treatment planning system. Measurement locations are denoted by dots. Dose difference values were interpolated between and extrapolated, when necessary, beyond measured locations.

The depth of dose max was found to be sufficiently similar to water (see Figure 5). The dosimetry test performed on the phantom showed that the mean difference (*x̄* ±  SD) between expected and received doses was 5.69% ± 4.56% for the standard bolus and 1.91% ± 1.31% for the custom device. The maximum difference from the expected dose was 15.1% for the standard and 3.51% for the custom bolus. The *p*‐value from the *t*‐test was 0.014, indicating a statistically significant difference between the custom and standard bolus. As shown by the data, areas of larger dose difference corresponded to areas with larger air‐gap. The percent difference from the expected dose for each location on the phantom is displayed in Figure 6. This figure shows that multiple locations on the phantom received radiation doses outside of 5% when using the standard bolus. In contrast, none of the locations received radiation doses that deviated by more than 5% when using the custom bolus. Furthermore, Figure 6 shows that the percent difference from the expected dose was lower in the custom than the standard bolus at 8 out of the 9 measured sites.

## 4 DISCUSSION

We found that the custom bolus reduced air‐gap and increased predictability of radiation dose delivered as compared to the standard bolus. Figure 4 demonstrates a clear difference between the conformability of the standard‐of‐care and custom bolus. Bolus customization has the potential to greatly reduce overall dosimetric uncertainties between the planned and delivered treatment. This is also demonstrated in Figure 6, where the custom bolus was able to adhere more closely to the expected dose. Though, it should be noted that since the dosimetric test was performed for a single AP field, these results likely represent a worst‐case scenario. Additionally, the conformability of the custom bolus is shown to decrease the variability of the bolus placement, which can impact overall dosimetric variability. As such, custom boluses could decrease the uncertainty of prescribed dose delivery of radiation therapy for superficial tumors.

It is also useful to note that this custom bolus workflow also has the potential to substantially reduce treatment set‐up time. During the patient study, the use of custom bolus took < 1 min compared to a standard Superflab setup of ∼4–5 min (between when the therapists pick up the bolus to being satisfied with the final placement), resulting in 80% reduction in time to correctly place the bolus, though this statistic was not repeatedly measured so further work would be needed to characterize time savings in a statistically robust way. However, based on this experience, there seems to be potential for time savings at each treatment session, which would mean that the patient would not have to wait as long for the RT to begin, reducing the potential of patient movement before treatment, while also increasing a clinic's linear accelerator time use efficiency.

In a similar study comparing a directly 3D‐printed a custom bolus to the standard, Robar et al. observed a decrease in setup time from 104 s to 76 s,^9^ supporting the idea that custom boluses may contribute to a more efficient clinic flow. The setup time in the study conducted here was more drastically reduced (∼75% reduction) than Robar et al. observed (∼27% reduction), potentially due to differences in the target region.

The sample size used in this study is the primary limitation. Additional testing with more patients would provide a more robust statistical characterization of the difference between these two methods. Additionally, the interpretation of the dosimetric test results is limited by the multiple uncertainties involved in obtaining these results, including uncertainty of the dose sampling process from the treatment planning system, as well as uncertainty in the measurement device itself.

Additionally, ∼2 months passed between the date that the patient CT simulation scan was collected and the date that the air‐gap CT scans for custom and standard boluses were taken. Examination of the alignment CT used for image‐guided RT showed tumor regression (∼1 cm maximal linear difference) over this time frame. However, we believe that this comparison for the purpose of air‐gap measurements is still fair as both the standard and custom bolus were designed using the planning CT, and all measurements were taken at the same time point. Additionally, if the patient CT scan for air‐gap calculations had been collected only days or weeks after the initial CT scan, the custom device may have reduced air‐gap by an even larger amount. However, this shows that even in cases where the patient morphology evolves during the course of treatment the custom bolus outperforms the clinical standard.

The decrease in air‐gap with use of the custom bolus rather than the standard may be at least partially attributable to the material used. The silicone rubber was malleable and slightly tacky, which allowed it to be pressed crevices even if they had changed slightly over the course of treatment. The silicone rubber used, while slightly malleable, is expected to maintain its cast shape throughout the lifetime of the device. This is aided by the use of the 3D‐printed shell from the silicone casting processing as a storage container. Additionally, the radiation distribution properties are not expected to change throughout the treatment duration, as supported by the results of preliminary testing mentioned in Section 2. However, future research will involve more extensive analysis over the entire course of the treatment regimen to fully validate the bolus fit, shape, and any relevant material changes.

There have been several studies that have sought to improve the treatment outcomes of RT using custom bolus devices by direct 3D printing of a custom bolus.^10^ 3D‐printed boluses have been shown to deliver 99.01% of the prescribed dose to the target.^11^ Additionally, Park et al. demonstrated that the difference from the prescribed radiation dose was no more than 3% with 3D‐printed boluses in six patients with breast cancer compared to the conventional bolus that had a maximum dose difference of 6%.^1^ It has been shown that standard bolus may result in significant dose differences from the calculated dose, while custom 3D‐printed boluses do not.^12^ Each of these studies helped to demonstrate that custom bolus would improve dose predictability compared to the clinical standard, improve conformity to the patient, and reduce air‐gaps compared to the clinical standard. There still remain improvements to be made to the 3D printing of bolus devices.

Our study sought to improve upon direct 3D printing of the bolus, which results in a much more rigid device than a silicone‐cast bolus. The rigid plastic may not conform to the patient's topography, and accounts less for day‐to‐day variations in the patient's surface. Canters et al. demonstrated that the theoretical dose received using a custom silicone bolus would be closer to the target dose than with the clinical standard.^6^ Park et al. demonstrated that a 3D‐printed, yet malleable, bolus for a phantom resulted in no air‐gap between the bolus and phantom surface.^13^ Our study supports these findings by showing that the custom silicone bolus conformed well to the target, reduced air gap, and increased dosimetric predictability.

While there is an existing tool that generate molds for boluses,^14^ they are limited to generating molds that fit within the print volume, which limits the treatment of larger areas and patients. Furthermore, these existing tools do not include sprues, increasing the likelihood of air bubbles persisting through the casting process, which would impair the radiation build‐up properties of the bolus. While the solution detailed in this bulletin does not currently tie into treatment planning software and may involve more steps than ordering directly from an existing company, it does offer a high degree of flexibility in customizing a bolus for scenarios not appropriately addressed by existing offerings.

## 5 CONCLUSION

In this study, we have demonstrated a technique to create a custom bolus that reduces air‐gap and increases predictability of dose on a phantom compared to the current standard. This may help to lead to an improved standard‐of‐care for RT patients. The dose delivery can be improved, thus potentially increasing treatment efficacy.

## CONFLICT OF INTEREST

The authors declare that there is no conflict of interest that could be perceived as prejudicing the impartiality of the research reported.

## AUTHOR CONTRIBUTIONS

Jonathan D. Tward was responsible for the initial study concept. All authors were involved in study design and data interpretations. Karissa M. Wang, Amanda J. Rickards, and Trevor Bingham were responsible for the entire equipment design and development process, with mentorship from Jonathan D. Tward and Ryan G. Price. All authors provided feedback on manuscript revisions and approved the final version.
